# Diagnosis and Management of Achalasia: Updates of the Last Two Years

**DOI:** 10.3390/jcm10163607

**Published:** 2021-08-16

**Authors:** Amir Mari, Fadi Abu Baker, Rinaldo Pellicano, Tawfik Khoury

**Affiliations:** 1 Department of Gastroenterology, Nazareth Hospital, Faculty of Medicine, Bar-Ilan University, Safed 16100, Israel; 2 Hillel Yaffe Medical Center, Department of Gastroenterology and Hepatology, Hadera 38100, Israel; fa_fd@hotmail.com; 3 Gastroenterology Unit, Molinette Hospital, 10126 Turin, Italy; rinaldo_pellican@hotmail.com; 4 Galilee Medical Center, Department of Gastroenterology, Faculty of Medicine in the Galilee, Bar-Ilan University, Safed 13100, Israel; tawfikkhoury1@hotmail.com

**Keywords:** dysphagia, achalasia, diagnosis, high resolution manometry (HRM), management, per oral endoscopic myotomy (POEM)

## Abstract

Achalasia is a rare neurodegenerative disorder causing dysphagia and is characterized by abnormal esophageal motor function as well as the loss of lower esophageal sphincter (LES) relaxation. The assessment and management of achalasia has significantly progressed in recent years due to the advances in high-resolution manometry (HRM) technology along with the improvements and innovations of therapeutic endoscopy procedures. The recent evolution of HRM technology with the inclusion of an adjunctive test, fluoroscopy, and EndoFLIP has enabled more precise diagnoses of achalasia to be made and the subgrouping into therapeutically meaningful subtypes. Current management possibilities include endoscopic treatments such as Botulinum toxin injected to the LES and pneumatic balloon dilation. Surgical treatment includes laparoscopic Heller myotomy and esophagectomy. Furthermore, in recent years, per oral endoscopic myotomy (POEM) has established itself as a principal endoscopic therapeutic alternative to the traditional laparoscopic Heller myotomy. The latest randomized trials report that POEM, pneumatic balloon dilatation, and laparoscopic Heller’s myotomy have comparable effectiveness and complications rates. The aim of the current review is to provide a practical clinical approach to dysphagia and to shed light on the most recent improvements in diagnostics and treatment of achalasia over the last two years.

## 1. Introduction

Achalasia originates from the Greek word a-khalasis, meaning lack of relaxation. It is characterized by a spastic lower esophageal sphincter and a lack of esophageal peristalsis resulting in esophageal outflow obstruction [1,2]. Achalasia is a rare disease, with an estimated incidence of 0.03 to 1.63 per 100,000 persons per year and a prevalence of 10 per 100,000 [1]. Achalasia is generally diagnosed between the third and sixth decades and affects both males and females at equal rates without racial predominance [3,4]. The natural history of achalasia is characterized by a chronic, life-long, but rarely life-threatening disease that seriously affects patients’ morbidity and quality of life [5]. When successfully treated, the quality of life almost returns to near normal for a long time; on the other hand, when untreated, the course is usually progressive, leading to esophageal lumen dilatation, which, over time, leads to a burned-out, decompensated sigmoid esophagus with its clinical related consequences, including malnutrition [5,6]. Longstanding achalasia is a significant risk factor for esophageal adenocarcinoma (50 folds) and esophageal squamous cell carcinoma, even when achalasia is adequately managed [7]. Nonetheless, no formal practical guidelines recommend endoscopic surveillance in achalasia patients. However, an endoscopy every three years is considered an acceptable practical surveillance approach for esophageal cancer in longstanding achalasia. In a follow up prospective study that included 32 achalasia patients after surgical treatment for achalasia, Ota and colleagues [8] reported that six patients (18%) developed esophageal cancer in a period of approximately 14.3 years after surgery. Therefore, continuing endoscopic surveillance is required for the detection of malignancy at an early stage. Special clinical awareness is further required in patients with other risk factors for esophageal cancer such as smoking, Barrett’s esophagus, alcohol drinking, and family history of esophageal cancer [9].

The main clinical presentations of achalasia are dysphagia, chest pain, vomiting, and weight loss. Despite its chronic course, these profoundly disturb a patient’s quality of life [6]. Not uncommonly, the diagnosis of achalasia may not be made for a long time; thus, a high level of clinical suspicion is needed. Esophageal dilation and sigmoid esophagus are considered serious structural consequences of untreated achalasia and eventually may lead to severe nutritional difficulties. Thus far, all treatment options target lower esophageal sphincter (LES) tearing, consequently allowing a bolus to pass through the esophago-gastric junction (EGJ) [6].

## 2. Medline Search

We performed a MEDLINE/PubMed search for achalasia. Articles discussing and reporting diagnosis, etiology and therapeutic options were extracted and fully accessed. Finally, we generated a comprehensive narrative review by summarizing the most updated data on the diagnosis and management of achalasia focusing on the latest updates from the last two years.

## 3. Etiology

The etiology of achalasia is still vague, and the precise pathogenesis mechanism of achalasia has been ambiguous up to now. Nevertheless, research findings propose a theory of autoimmune origin, leading to a cascade of a destructive inflammatory processes resulting in destruction of the nitric oxide releasing neurons within the myenteric plexus and the vagus nerve fibers of the lower esophageal sphincter [7]. In end-stage disease, this affects the cholinergic neurons and subsequently progresses to the loss of inhibitory neurons containing nitric oxide synthase and vasoactive intestinal peptide A. This leads to an impaired relaxation of the lower esophageal sphincter [10]. Several patho-mechanisms were proposed as possible triggers of this immuno-destructive process, including underlying viral infection [11], idiopathic autoimmune trigger, and genetic predisposition [12]. Recent data have further addressed the role of autoimmunity and viral infection as the trigger for achalasia development. Innate immune system cells, including eosinophils and mast cells, have been increasingly observed in the esophageal tissue of achalasia patients [13,14,15,16]. These cells are already described as important mediators of immune-mediated inflammation and in degenerative neurological diseases [17]. Several studies have reported the involvement of the innate immune system in the pathogenesis of achalasia [13,14,18,19,20]. Moreover, the adaptive immune (B and T cells) system has recently been shown to play a major role in the development of achalasia. Previous studies using immunohistochemical analysis have shown a strong infiltration of CD3^+^ T lymphocytes within the esophageal mucosa of achalasia patients, thereby causing myenteric plexitis [21,22]. One recent study showed an increased expression of T lymphocytes (Th22, Th 17, Th 2, Th1, and T regulatory cells) in the lower esophageal sphincter tissue of achalasia patients [23,24]. Additionally, other studies have addressed the emerging role of proinflammatory cytokines (interleukin (IL)-22, IL-17, interferon-gamma, IL-6, and tumor necrosis factor alpha) that were overexpressed in achalasia patients compared with controls [23,25]. However, still more studies are needed to explore the dominant immune cells and cytokines that trigger the development of achalasia and to determine the underlying trigger for the activation of those immune cells and pathways [26]. Still, an underlying viral infection is an acknowledged and reported factor behind achalasia development [27,28]. Based on the existing evidence, the most known viral infections that are associated with achalasia are the herpes virus family (Herpes simplex virus, Epstein–Barr virus, Varicella Zoster virus, and Cytomegalovirus) [29,30], Paramyxoviruses [31], and human immunodeficiency virus (HIV) [32]. In the last few years, evolving new theories have been reported that attempt to address the etiological mechanisms of achalasia, starting from the involvement of the innate immune system. These include mast cells and eosinophils that reach the adaptive immune system and the cytokines that directly induce inhibitory neurons and damage the esophageal muscle layer. Furthermore, studies on the potential role of viral infection in achalasia cannot be ignored. All proofs lead to the conclusion that viruses may lay the foundation for autoimmune responses that attack inhibitory neurons.

## 4. Diagnostic Approach to Dysphagia and Achalasia

Dysphagia is considered an alarm symptom that mandates the performance of esophago-gastro-duodenoscopy (EGD) as an initial diagnostic modality to exclude structural or mucosal lesions in the esophagus or the stomach cardia. Examples of these include tumors, inflammation, esophageal rings, strictures, and other pathologies that can mimic achalasia, a condition traditionally named pseudochalasia 4. A clinical suspicion of pseudo-achalasia should be sought in patients older than 55 years of age with a prompt onset of solid dysphagia that proceeds to liquid dysphagia and weight loss [33,34]. Classic endoscopic findings of achalasia present in about half of the cases include widening of the esophagus, residue in the esophageal lumen, and obstructed EGJ.

An additional important diagnosis is eosinophilic esophagitis (EoE), an immune-mediated/allergic disorder involving the esophagus causing dysphagia and diagnosed by eosinophils predominant inflammation [35]. Multiple biopsies are mandatory to confirm the diagnosis. Indicative endoscopic findings of EoE include mucosal thickening and edema, ring formation, and white patchy exudates and fibrosis in the late stage [35]. After the exclusion of anatomical, structural, and inflammatory conditions, HRM study is necessary to assess the esophageal motor function and the relaxation of the lower sphincter.

## 5. High-Resolution Manometry and the Chicago Classification Version 4.0

**Figure 1:**
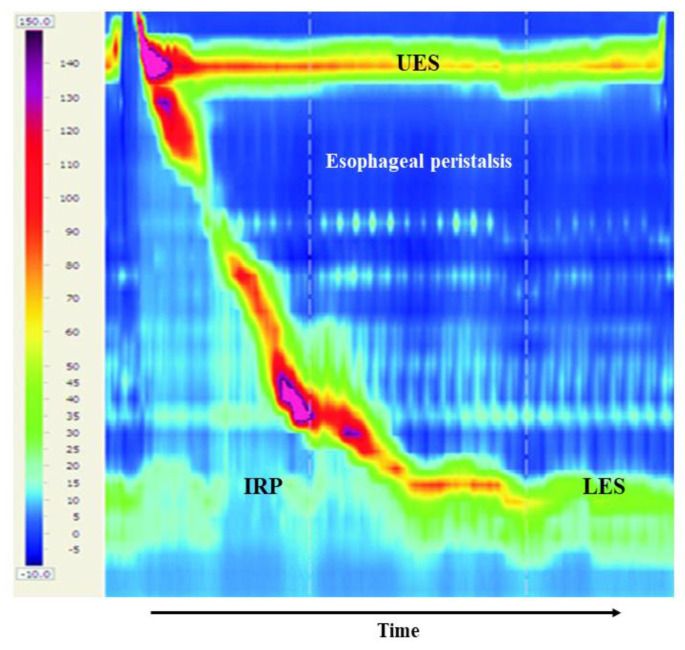
A five milliliter water swallow starts with the opening of the upper esophageal sphincter (UES). One normal esophageal peristalsis and normal lower esophageal sphincter (LES) relaxation is shown. The LES relaxation is measured over a 10 s period, as shown in the box, by measuring the median integrated relaxation pressure (IRP) (supplied from the gastroenterology department at EMMS Nazareth hospital).

High-resolution manometry (HRM) is the most accurate investigative system ordinarily utilized in order to study esophageal motility and the LES function when evaluating upper gastrointestinal symptoms including dysphagia when endoscopic and radiologic modalities do not elucidate their cause [36]. The HRM catheter includes 36 pressure sensors disseminated thoroughly over the catheter. The probe is gently entered through the nose and crosses the esophageal body up to the LES. The pressure sensors register pressure changes throughout the swallowing process, and the collected records are processed in a dedicated program that converts these data into a colorful spatiotemporal scheme, where variations in pressure produced by esophageal peristalsis are showed as color distinctions throughout the study duration (Figure 1). The addition of impedance measurement to the HRM studies has enabled impartial valuation of the esophageal clearing capability of fluids to the stomach [33].

The Chicago classification, currently in its fourth version (CCv4.0), is a conceptualized and standardized approach to interpreting HRM findings [10]. One major improvement of the CCv 4.0 is providing rigorous definition and diagnosis for various manometric findings and highlighting their clinical significance and relevance. Conveyed by the expanding knowledge and experience with HRM, the CCv4.0 aimed to provide an updated classification scheme as well as to apply a more rigorous standardized HRM protocol, with the inclusion of provocative tests aiming to reproduce the natural behavior of drinking and eating. These tests include water swallows in various positions, setting and supine; the multiple rapid swallow test (MRS), a repetitive and rapid swallow of water that assesses the deglutitive inhibition function of the LES; and the rapid drink challenge of 200 mL water (RDC), which assesses the deglutitive inhibition function of the LES in addition to depicting the ‘recovery capability’ of the esophagus by the production of a powerful clearing swallow. Additionally, the addition of bread swallows or a test meal is optional when there is high suspicion of EGJ outflow obstruction (EGJOO). The inclusion of provocation or ‘adjunctive tests’ in the HRM protocol is based on the fact that the standard 5 mL water swallow is unrepresentative of the normal esophageal physiology and performance, infrequently induces esophageal symptoms, and might under-diagnose motility disorders of clinical significance. The previous Chicago classification version 3 (CCv3.0) included only 10 mL water swallows in the supine position [37]. However, many studies suggested that adjunctive tests could improve the diagnostic yield of HRM for the detection of motility disorders and for better defining the clinical relevance of these findings [38,39,40,41,42]. Furthermore, to ease the implementation of solid swallows in routine clinical practice, Hollenstein et al. performed a development and validation study that aimed to define normal values of esophageal metrics for solid swallows. The authors developed a classification of motility disorders (named the Chicago classification) and this was adapted for solids. The developed classification was applied in the assessment of patients (750) with esophageal symptoms, and the authors confirmed that the inclusion of a solid swallow test improved the diagnosis of clinically pertinent esophageal motility disorders in a cohort with dysphagia and reflux symptoms [43].

**Figure 2:**
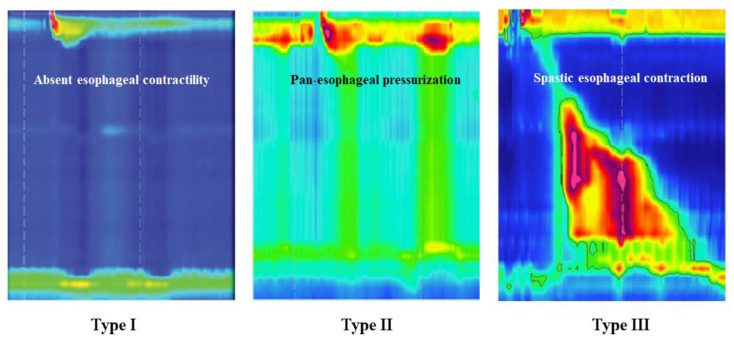
The three achalasia subtypes determined by the Chicago classification (supplied from the gastroenterology department at EMMS Nazareth hospital).

Since the introduction of the Chicago classification, three subtypes of achalasia could be identified, depending on the esophageal peristalsis failure type. According to the last Chicago classification (CCv4.0), achalasia type I is defined as an increased intergrade relaxation pressure (IRP—an indicator of the relaxation capability of the LES) and the complete absence of esophageal contractility (totally failed peristalsis with loss of LES relaxation). Type II achalasia is characterized by the production of ‘pressure columns’ due to pan-esophageal pressure through the hollow esophagus. According to the CCv4.0, type II achalasia is defined as an elevated IRP associated with defective esophageal peristalsis (pan-esophageal pressure in at least 20% of swallows). Achalasia type III is characterized by the presence of premature and/or spastic contractions and a conclusive diagnosis is obtained through the detection of an elevated IRP and the presence of at least 20% premature contractions (Figure 2). This subtyping has improved our understanding of achalasia and, furthermore, has influenced the management plan, enabling a more personalized therapeutic approach. Functional or idiopathic EGJOO—previously called “variant achalasia”—is a disorder more commonly encountered than achalasia and specified by normal esophageal contractility alongside distal obstruction at the level of the LES. Possible etiologies for functional EGJOO include a true idiopathic failure of the LES to relax (a condition that could be treated as achalasia) or the result of technical issues such as the patient’s position or the angulation of the catheter [39,44,45,46]. Secondary causes of outflow obstructions include mucosal or submucosal lesions, EOE, external compression, strictures, post-surgical complications, as well as medications such as opioids [47,48,49] (Table 1). Treatment should target the source of the outflow obstruction, such as surgical corrections of anatomical abnormalities, endoscopic dilation of strictures, EOE management, and opioid cessation [36].

## 6. Barium Swallows

Barium esophagography has commonly been used to evaluate esophageal morphology prior to surgery. Recently, the timed barium swallow (TBS) has been used to assess treatment success by evaluating esophageal emptying. Measurement of the retained barium column at several time points after the swallow has been accepted as a reliable tool to objectively assess the level of obstruction at the esophagogastric junction. Moreover, barium emptying studies have gained special popularity in the post treatment period, and they correlate well with treatment response [50]. TBS has several advantages: it is simple, practical, reproducible, economic, non-invasive and well-tolerated by patients. A latest work by Sanagapali et al. that aimed to study the role of barium surface area compared with the traditional barium column as an indicator of treatment response revealed that barium surface area decrease predicted a more precise treatment response [51].

## 7. EndoFLIP

Endoflip is a novel diagnostic device that permits measurement of the level of distensibility at the esophagogastric junction as well as is capable of detecting the various achalasia subtypes with a high level of confidence and accuracy, particularly with the advent of combining distensibility sensors to manometry sensors [52]. The test is performed while the patient is sedated, eliminating the inconvenience related to HRM and potentially replacing it in select patients.

## 8. Treatment of Achalasia

The most fundamental goals of treating achalasia are to attain symptomatic relief and to improve patients’ quality of life and work capability. Since the repair and the rehabilitation of the defective contractility are impractical and unrealistic, the eventual target of treating achalasia is to release the resistance at the esophagogastric junction. This treatment choice is not straightforward, and a personalized approach should be adopted that takes into account factors including the demographics and medical background of the patient, the achalasia subtype, and the patient’s predilection (Table 1) [53]. Importantly, when describing treatment outcomes in achalasia, most previous trials relied on subjective symptom relief as reported by patients, generally by applying the EKARDT score. The EKARDT score includes the four main achalasia symptoms of dysphagia, regurgitation, weight loss, and chest pain. The score points relied on the frequency of each symptom reported by patients and ranges from 3 to 12 (worst symptoms) [54,55]. Nonetheless, despite the widespread implementation of the EKARDT score in clinical practice, it has not been validated yet for this purpose (I). Moreover, most trials considered an Eckardt score of >3 or a reduction in symptoms of <50% as treatment failure. However, several limitations exist with this instrument of assessing treatment outcomes, including using subjective symptoms that could be perceived differently between patients. It can be also be misleading, frequency and time intervals of applying the EKARDT core have yet to be defined, and the cardinal achalasia symptoms could be provoked by pathologies other than achalasia.

Botulinum toxin (Botox) is a well-known therapeutic option for achalasia that has been used for decades [56]. When injected into the distal esophagus and to the LES, the toxin inhibits the release of acetylcholine, which eventually leads to a transitory inhibition of the contractility of LES smooth muscle fibers. Despite the excellent safety profile of Botox injection, the key drawback of this therapeutic option is its short-term durability given a substantial decline in symptoms relief after 6 and 12 months [56]. Therefore, Botox use is restricted to special cases such as comorbid elderly patients or as a temporarily relief before surgery, POEM, or balloon dilation. 

Pneumatic balloon dilation is a therapeutic option where a pneumatic balloon is placed in the LES under the guidance of fluoroscopy. The gradual inflation of the balloon leads to mechanical disruption of the LES and relieves the obstruction at the esophagogastric junction. Currently, the preferred protocol for dilation is using a graded attitude, where dilation starts with the 30 mm balloon but the balloon diameter increases in subsequent sessions to 35 mm and up to 40 mm. The gradual dilation approach has been shown to have greater efficacy and a higher safety profile [57]. Pneumatic balloon dilation is long-lasting, with a symptomatic relief over 80% after 2 and 5 years [58]. The long-standing clinical success of pneumatic balloon dilation after 2 and 5 years is satisfactory and similar to surgical outcomes [59]. Complications related to balloon dilation are rare and may include the development of esophageal reflux symptoms in 15–35% of patients. Esophageal perforation is rare and occurs in about 2% of cases and very rarely leads to bleeding [60].

Heller myotomy is an well-established procedure for achalasia treatment that has been performed for more than a century and involves the dissection of the LES smooth muscle fibers. The incidence of esophageal reflux symptoms and the development of erosive esophagitis after the myotomy have been significant; therefore, surgeons also complete a partial fundoplication wrap of the posterior (Toupet) or the anterior (Dor) to prevent reflux symptoms and complications. LHM is a safe and effective therapeutic modality with durable symptomatic relief, estimated to be over 85% after 5 years [59].

Recently, POEM has been acknowledged as an efficient novel treatment modality with an excellent safety profile [61]. This procedure is performed by an invasive gastroenterologist or a surgeon and normally in an operating room while the patient is under sedation and intubated. The procedure involves the creation of a tunnel located at the submucosa using an endoscopic knife. The tunnel generally originates at the middle or lower esophagus and ends up at the stomach cardia, commonly 2–3 cm beyond the LES. Endoscopic myotomy is completed by either anterior or posterior dissection of the circular muscle’s fibers. Ujiki et al. performed a pooled data analysis of three comparative studies that aimed to assess various clinical outcomes of POEM and LHM. The analysis revealed comparable results of therapeutic options including success rate, complications, perforation rate, as well as procedure time [62]. Kumbhari et al. performed a comparative study over 75 patients and showed that POEM seems to be a better therapeutic option for achalasia type III when compared with LHM [63]. Even though it is suggested that there is an increased trend towards the development of GERD after POEM, when looking carefully at the reported results, most reflux esophagitis cases were mild and responded well to conservative treatment with proton pump inhibitor drugs. However, the main difficulty of the POEM technique is the operator learning curve; a recent study reported that mastering POEM in Latin America requires approximately 61 procedures both for POEM efficiency and to accomplish the procedure within 97 min [64]. Another previous study reported that 40 POEMs are needed to achieve efficiency and that 60 POEMS are needed to attain mastery [65].

Lastly, surgical esophagectomy is a radical procedure that is considered a last resort therapeutic option for longstanding advanced achalasia cases, which are rarely encountered and estimated to occur in 2–5% of cases. End-stage achalasia generally involves pathological dilation of the esophageal tubular structure and that even could be associated with alterations of the esophageal morphology (creating a sigmoid shape or mega-esophagus). Even though LHM could still be considered and performed successfully in end-stage achalasia with morphologic distortion of the esophagus, the radical esophagectomy might be the final possibility to improve patients’ nutritional status, to ease their symptoms, and to improve their general performance and quality of life. Notably, esophagectomy is a major chest surgery and is associated with high adverse events including hospital-acquired pneumonia in 10% of cases, leaks at the surgical site causing chest infections in about 7% of cases, and finally, a mortality risk of 2% [6].

## 9. Treatment Decision-Making and Predictors of Outcomes

Recent published data from retrospective, prospective, and randomized studies indicate that there is no superiority between the three options of pneumatic dilation, LHM, and POEM. Considerations regarding the choice of therapy are largely determined by the achalasia subtype; clinical presentation; patient’s age and fitness; the available expertise; and importantly, the patient’s preferences. Significantly, the emerging data from the last decade points toward an association between the achalasia type and post-therapy clinical outcomes [43]. A post-hoc analysis by Rohof et al. of the European achalasia registry study found an association between achalasia subtype and treatment outcomes; the efficacy of pneumatic dilation was outstandingly excellent in type II achalasia but significantly decreased to 40% in type III achalasia [66]. Conversely, Kumbhari et al. conducted a multicenter comparative study that aimed to evaluate the treatment outcomes of 75 patients with type III achalasia and showed an excellent treatment success (98%) following POEM in comparison to 80% after LHM during both short-term (8.6 months) and long-term (21.5 months) (*p* < 0.01) follow-up [63]. This added value of POEM over LHM is logically explained by the capability of the endoscopic approach to achieve a tailored proximal extension of the myotomy. Oude and colleagues conducted a systematic review and meta-analysis that aimed to study various factors that might be associated with achalasia treatment outcomes. The analysis included 75 published studies and revealed that only the manometric pattern of achalasia and patient age were recognized as the most applicable predictors of clinical response [67].

## 10. Conclusions

Achalasia is an long-standing disease that has attracted much interest in the last two decades due to the revolutionary progress in its understanding and management. The introduction of the HRM with impedance along with the construction of the Chicago classification and their implementation in clinical practice has profoundly enriched our understanding of the esophageal and LES functions and has eventually led to classifying achalasia into three different types based on diverse manometric patterns. Moreover, EndoFLIP and the improved methodology in barium studies added to our knowledge, and these modalities complement HRM in specific clinical scenarios. The introduction of the POEM to the therapeutic arsenal has drastically reformed the attitude to achalasia therapy. The POEM procedure seems to be promising, with outcomes comparable to conventional procedures such as LHM and pneumatic balloon dilation. Nonetheless, more prospective studies are required to properly determine the long-term efficacy and safety of POEM. The treatment choice of achalasia should be tailored, taking into account several clinical and manometric factors.

## Figures and Tables

**Table 1:** Achalasia subtypes.

Achalasia		
	Manometric Findings	Treatment *
Type I (classic)	Non-relaxing LES and absent peristalsis	LHM, PD, POEM
Type II (pan-esophageal pressurizations)	Non relaxing LES and pressurization	LHM, PD, POEM
Type III (spastic)	Non-relaxing LES and spastic contractions	POEM

* Botox injection use is limited to elderly, frail patients and could be offered to all achalasia subtypes. LES: Lower esophageal Sphincter; LHM: Laparoscopic Hiller Myotomy; PD: Pneumatic Dilation; POEM: Per-Oral Endoscopic Myotomy (POEM).

## Abbreviations

LES	Lower esophageal sphincter
EGJ	Esophago-gastric junction
EGJOO	Esophago-gastric outflow obstruction
HRM	High-Resolution Manometry
POEM	Per oral endoscopic myotomy
EOE	Eosinophilic Esophagitis
EGD	Esophago-Gastro-Duodenoscopy
LHM	Laparoscopic Heller myotomy
RDC	Rapid drinking challenge
MRS	Multiple rapid swallows
TBS	Timed barium swallow
